# Differentiating white matter measures that protect against vs. predispose to bipolar disorder and other psychopathology in at-risk youth

**DOI:** 10.1038/s41386-021-01088-1

**Published:** 2021-07-20

**Authors:** Renata Rozovsky, Amelia Versace, Lisa K. Bonar, Michele Bertocci, Cecile D. Ladouceur, Jay Fournier, Kelly Monk, Halimah Abdul-waalee, Genna Bebko, Danella Hafeman, Dara Sakolsky, Tina Goldstein, Boris Birmaher, Mary L. Phillips

**Affiliations:** 1 Department of Psychiatry, University of Pittsburgh School of Medicine, University of Pittsburgh, Pittsburgh, PA USA; 2 Magnetic Resonance Research Center, Department of Radiology, University of Pittsburgh Medical Center, University of Pittsburgh, Pittsburgh, PA USA

**Keywords:** Bipolar disorder, Prefrontal cortex

## Abstract

Bipolar disorder (BD) is highly heritable. Identifying objective biomarkers reflecting pathophysiological processes predisposing to, versus protecting against BD, can help identify BD risk in offspring of BD parents. We recruited 21 BD participants with a first-degree relative with BD, 25 offspring of BD parents, 27 offspring of comparison parents with non-BD psychiatric disorders, and 32 healthy offspring of healthy parents. In at-risk groups, 23 had non-BD diagnoses and 29, no Axis-I diagnoses(healthy). Five at-risk offspring who developed BD post scan(Converters) were included. Diffusion imaging(dMRI) analysis with tract segmentation identified between-group differences in the microstructure of prefrontal tracts supporting emotional regulation relevant to BD: forceps minor, anterior thalamic radiation(ATR), cingulum bundle(CB), and uncinate fasciculus(UF). BD participants showed lower fractional anisotropy (FA) in the right CB (anterior portion) than other groups (*q* < 0.05); and in bilateral ATR (posterior portion) versus at-risk groups (*q* < 0.001). Healthy, but not non-BD, at-risk participants showed significantly higher FA in bilateral ATR clusters than healthy controls (*q*s < 0.05). At-risk groups showed higher FA in these clusters than BD participants (*q*s < 0.05). Non-BD versus healthy at-risk participants, and Converters versus offspring of BD parents, showed lower FA in the right ATR cluster (*q*s < 0.05). Low anterior right CB FA in BD participants versus other groups might result from having BD. High bilateral ATR FA in at-risk groups, and in healthy at-risk participants, versus healthy controls might protect against BD/other psychiatric disorders. Absence of elevated right ATR FA in non-BD versus healthy at-risk participants, and in Converters versus non-converter offspring of BD parents, might lower protection against BD in at-risk groups.

## Introduction

Bipolar Disorder (BD) is one of the most debilitating psychiatric illnesses, affecting 1–3% of the adult population worldwide. BD commonly occurs in adolescence or early adulthood, and causes emotional and behavioral problems often leading to poor psychosocial function, substance abuse and suicide [1–3]. BD is highly heritable [4–7]. Unfortunately, BD is frequently misdiagnosed or diagnosed late [8]. Therefore, earlier identification of future BD risk is a key research priority in BD [9]. The longitudinal Bipolar Offspring Study (BIOS) [10], which examines the development of psychiatric symptomatology in offspring of Bipolar parents (OBP), and other studies reported rates of BD in OBP ranging from 15 to 20% [11–13]. In addition, in offspring of parents with early-onset BD, the risk increases to 30–50% [5, 14, 15]. Thus, pediatric populations, and especially OBP, are appropriate populations in which to examine factors associated with future BD risk.

Identifying objective biomarkers that reflect pathophysiological processes and are not dependent on subjective report is an important step to facilitate earlier and accurate identification of future BD risk. Structural and/or functional abnormalities in neural circuitries important for processes that are aberrant in BD, e.g., emotional regulation and reward processing [16] might be apparent before the onset of symptoms in youth at risk of BD [17]. Thus, neuroimaging studies of OBP are a promising way to identify objective markers of future BD risk in a potentially vulnerable population.

Yet, given that BD-parents often show high rates of comorbid psychopathology (e.g., depression, ADHD), OBP are at increased risk not only for BD but also for other psychopathology, especially depression, ADHD, anxiety, and substance abuse [10, 18, 19]. As a result, it is difficult for neuroimaging studies of OBP to differentiate neural markers associated with non-BD psychopathology from the more specific markers associated with BD risk in OBP. Other factors that complicate the identification of BD risk factors in OBP are that prodromal symptoms of BD, including non-BD disorders, in OBP often overlap with those in offspring at risk of other psychiatric disorders, due to OBP having parents with these other disorders [20, 21]. Furthermore, living with a parent with any psychiatric disorder also likely affects the offspring’s development and general well-being [22–24]. More recent BIOS studies [25, 26] have therefore included offspring of comparison parents with non-BD psychopathology (OCP) and healthy offspring of healthy parents, to differentiate neuroimaging abnormalities common to OBP and OCP that are more likely to be associated with risk for non-BD disorders from those that are specific to OBP, and thus more likely to be associated with risk for BD. Another difficulty with neuroimaging studies of OBP is that even after including OCP, it remains unclear whether any abnormalities that are specific to OBP reflect risk for future BD or protective markers against the development of BD and other psychiatric disorders, as no studies of OBP (and OCP) to date included a comparison group of offspring with BD.

White matter (WM) abnormalities are increasingly implicated in the neurobiology of BD in diffusion imaging (dMRI) studies [27–31]. These abnormalities might result from aberrant maturation [32]. Lower and higher fractional anisotropy (FA), an indirect measure of fiber collinearity in WM tracts, are reported in BD, with abnormalities in adult and pediatric BD cohorts observed in networks supporting emotional regulation, including the corpus callosum, cingulum bundle (CB), anterior thalamic radiation (ATR), and uncinate fasciculus (UF) [32–50]. Abnormalities in WM in adults and youth at-risk for BD versus healthy controls have also been reported [25, 32, 46, 51–55]. Thus, examining WM in OBP, OCP, OHP and offspring with BD can help differentiate biomarkers of future BD risk from biomarkers conferring risk for other psychiatric disorders, and from biomarkers conferring protection against future BD and other psychiatric disorders.

To further understanding of BD risk, we therefore recruited four offspring groups: offspring of BD parents with BD themselves (BD), offspring who did not have a BD diagnosis themselves of BD parents or of comparison parents with non-BD psychopathology (OBP and OCP, respectively), and healthy offspring of healthy parents (OHP). This allowed us to: 1. identify abnormalities (relative to OHP) in WM tracts of interest in at-risk offspring (OBP, OCP) that resembled abnormalities in offspring with BD (i.e., disease biomarkers), and were thus more likely to reflect biomarkers of BD risk versus risk for other psychiatric disorders or biomarkers conferring protection against BD and other psychiatric disorders; and 2. identify abnormalities (relative to OHP) in WM tracts in healthy OBP and OCP versus non-BD OBP and OCP, to help differentiate between biomarkers of protection against, versus risk for, BD and other psychiatric disorders. Additionally, we included five participants (four OBP, one OCP) who developed BD after their neuroimaging assessment. Even if underpowered, the inclusion of this subgroup allowed us to explore which, if any, WM abnormalities in these individuals resembled the abnormalities in BD offspring, as an additional step to elucidate the specific WM abnormalities that might predispose to BD. We also used tractometry [56] to determine whether the main effects of group were focal or across the entire tract.

Given the extant literature in BD, we focused on the ATR, CB, forceps minor, and UF. We hypothesized that:

1. BD would show abnormally reduced FA in all WM tracts of interest versus other groups, reflecting BD disease biomarkers.

2. OBP and OCP would show abnormally reduced FA in WM tracts showing abnormally reduced FA in BD, reflecting BD risk biomarkers; and these abnormalities would be of significantly greater magnitude in OBP than OCP, and in non-BD OBP and OCP versus healthy OBP and OCP.

3. Healthy OBP and OCP would show abnormalities in WM tracts that were not shown by non-BD OBP and OCP, reflecting biomarkers conferring protection against BD and other psychiatric disorders.

4. In exploratory analyses, FA abnormalities in OBP and OCP that were also common to BD, BD risk biomarkers, would be present in the small group of OBP and OCP that converted to BD after the neuroimaging assessment.

Previous findings did not allow us to make hypotheses about the more specific nature of the above patterns of WM abnormalities.

## Methods and materials

### Participants

121 OBP, OCP, and OHP aged 8–17 years, were recruited from BIOS [8]. BD had a confirmed BD diagnosis and at least one 1st/2nd degree relative with a confirmed BD diagnosis (Structured Clinical Interview for DSM-IV (SCID) [57] or Family History questionnaire (Supplementary Materials)). OBP had no BD diagnosis and had at least one parent with a BD diagnosis (SCID). OCP had no BD diagnosis and had at least one parent with a non-BD diagnosis, including Major Depressive Disorder, Attention-Deficit/Hyperactivity Disorder, and/or an Anxiety Disorder (*n* = 25); or had themselves an Anxiety Disorder or Depressive disorder (*n* = 2). OHP were healthy offspring with no family history (1st degree) of psychiatric illness, other than mild symptoms of anxiety disorders/PTSD in parents (*n* = 2). For exclusion criteria and sensitivity analysis, see Supplementary Materials.

Eleven participants were excluded from analyses due to movement and/or poor data reconstruction. Based on clinical assessment and interviews with offspring and their parents, the final sample (*n* = 110) included four groups: BD—offspring with BD of parents with BD (*n* = 21, females/males = 14/7, mean age [SD] = 13.89 [2.89]); OBP—offspring without BD of BD parents (*n* = 25, females/males = 12/13, mean age [SD] = 13.59 [2.49], 1 left-handed), OCP –offspring without BD of parents with non-BD disorders (*n* = 27 females/males = 12/15, mean age [SD] = 13.12 [2.24], 1 left-handed), and OHP –healthy offspring of healthy parents (*n* = 32, females/males = 14/18, mean age [SD] = 13.19 [2.57], 2 left-handed).

All offspring were clinically assessed every two years after intake. Five offspring (four OBP and one OCP) converted to BD after scan: Converters (*n* = 5, females/males = 2/3, mean age [SD] = 15.05 [2.57]). The mean time between scan and BD conversion was 3.05 years (SD = 1.64). The mean time between the first scan and the latest follow-up for OBP and OCP was 5.2 years (SD = 1.74). Before study participation, parents and guardians provided written informed consent, and youth provided written informed assent. The Pittsburgh University IRB approved the study.

### Clinical assessment

Psychiatric diagnoses were confirmed by a licensed psychiatrist or psychologist before the neuroimaging assessment, using the Kiddie Schedule for Affective Disorders and Schizophrenia for School-Age Children (K-SADS)—Present and Lifetime Version [58] for offspring (inter-rater reliability for K-SADS is 0.80), and the Structural Clinical Interview for DSM-IV [59] and Family History Screen [60] for parents. Assessments also included the Screen for Child Anxiety Related Disorders (SCARED) [61, 62], Children’s Affective Lability Sale (CALS) [63], Mood and Feelings Questionnaire (MFQ) [64], to assess depressive symptoms, K-SADS Mania Rating Scale (KMRS) [65], and K-SADS Depression Rating Scale (KDRS) [58]. Parent- and child-reported SCARED, CALS, and MFQ were administered on the scan day. K-SADS Mania Rating Scale (KMRS) [65], and K-SADS Depression Rating Scale (KDRS) [58] were administered to parents and offspring on average 2 months before or after the scan, at the alternate yearly assessments: range from 1 to 475 days; Mean = 66.7 and SD = 72.8. Regression analysis revealed no significant effects of K-SADS latency on our main findings (Supplementary Materials).

All participants also completed medication forms that documented psychotropic medications used at the time of the scan. Sixteen BD participants were taking psychotropic medications (stimulants, antipsychotics, antidepressants, benzodiazepines, mood stabilizers). Two OBP participants were taking psychotropic medications for non-BD diagnoses (one was taking a stimulant, and one a non-stimulant). Three OCP were taking psychotropic medications for non-BD diagnoses (two participants were taking antidepressants and one was taking stimulants). Handedness was assessed using the Annett Behavioral Handedness Index [66]. Interviewers also evaluated socioeconomic status (SES) [67].

### dMRI data analysis

For diffusion imaging acquisition, please see Supplementary Materials. Diffusion-weighted images were transferred to a Unix-based workstation. Distortions due to eddy currents and head motion were reduced using *eddy correct* [68] within the Functional Magnetic Resonance Imaging of the Brain Software Library (FSL; http://www.fmrib.ox.ac.uk/fsl). The gradient vectors were rotated accordingly to the spatial transformations applied to their corresponding volumes using *fdt_rotate_bvecs* within FSL. Skull and non-brain voxels were stripped using the brain extraction tool (bet) [69] within FSL [70]. Tracts were segmented in native space using TractSeg [56], using a convolutional neural network-based approach (Tract Orientation Mapping) to automatically segment 50 major WM tracts using the field of fiber orientation distribution function (fODF), as implemented in mrtrix-3 [71]. TOM facilitates bundle-specific tractography based on a learned mapping from the original fODF peaks to a list of tract orientation maps [72]. TractSeg allows for a point-wise tractographic approach (tractometry). The reconstructing algorithm used was Constrained Spherical Deconvolution. After accurate reconstruction of the tracts, the main dMRI metric of interest, FA, was extracted in 98 nodes along each tract (point-wise values) and averaged across the entire tract (mean values) and exported into SPSS (version 26). Given that other dMRI metrics [73], i.e., radial diffusivity (RD), an indirect measure of fiber dispersion and possibly abnormal myelination, and axial diffusivity (AD), the magnitude of the principal diffusion direction of the fibers, can inform understanding of FA abnormalities, these additional metrics were extracted from clusters showing a main effect of group on FA.

### Statistical analysis

Age [74, 75], sex [76], and IQ [77, 78] on WM, these variables, and handedness, were covariates in all analyses of dMRI measures. To test our hypotheses, a threefold analytic approach was applied. A false discovery rate (FDR; *q* < 0.05) correction [79] was used to account for multiple comparisons in level 2 and 3 analyses below.

### Between-group differences in demographic and clinical measures

Chi-squared tests for sex, handedness, and differences in the proportion of offspring with non-BD diagnoses in each group were performed. To examine differences between groups in age, IQ (assessed by Wechsler Abbreviated Scale of Intelligence [80], SES (socioeconomic status by Hollingshead [67]), SCARED, CALS, MFQ, KMRS, and KDRS, nonparametric Kruskal–Wallis and Dunn’s post hoc tests were performed, as appropriate.

### 2. Main effect of group on FA in WM tracts of interest

*2.i. Multivariate Analysis of Variance (MANOVA)* was first performed to identify the main effect of group (BD, OBP, OCP, OHP) on mean FA in the seven tracts of interest: forceps minor, and three bilateral tracts: ATR, CB, UF. Group was the independent variable; age, sex, IQ, and handedness were covariates; and mean FA in the 7 tracts was the multiple dependent variables.

*2.ii. Univariate analyses* (seven parallel ANCOVAs; one for each tract of interest) were performed to identify which tract contributed to the main effect of group across all tracts of interest. Group and covariates were the independent variables, and mean FA in each tract was the dependent variable.

*2.iii. Tractometry analyses* [56] determined whether the main effects of group were focal or across the entire tract. To identify the specific nodes that contributed to the main effect of group, 98 parallel ANCOVAs (one for each node [see dMRI data analyses above] with group as the main independent variable) were performed for each tract showing a main effect of group in *2.ii*. FDR correction (*q* ≤ 0.05) was performed across all such ANOCVAs for nodes in tracts showing the main effect of group in *2.ii*. Cluster (s) comprising 10 or more nodes in each tract that showed a significant main effect of group in 2.ii were identified. FA was averaged across all nodes in the cluster (s) for further analyses in *3 below*.

*RD and AD*. To interpret the main between-group FA findings, mean RD and AD were extracted from the same cluster (s) identified in *2.iii* for further analyses.

### 3. Between-group differences in clusters

*3.i. Pair-wise comparisons among BD, OBP, OCP, and OHP*. To test our main hypotheses, 12 pair-wise comparisons (3 for each group: BD, OBP, OCP, OHP) were performed to determine which specific between-group comparisons were driving the main effect of group in *2.iii*. Here, pair-wise comparisons were performed using FA in cluster (s) identified in *2.iii*. Age, sex, IQ, and handedness were covariates. An FDR correction (*q* < 0.05) accounted for multiple comparisons.

*3.ii. Pair-wise comparisons between BD, non-BD OBP and OCP, healthy OBP and OCP, and OHP. 12* pair-wise comparisons (3 for each group) were also performed to determine the extent to which having a non-BD diagnosis contributed to the effects in *3.i*, using *q* < 0.05.

*3.iii. Converters*. To further determine whether Converters resembled offspring with BD or other offspring groups, pair-wise *t* tests explored differences between Converters and other groups (BD, OBP, OCP, OHP) in each cluster showing the main effect of group in 2.iii, using FDR *q* < 0.05.

Exploratory analyses examined effects of medication, and relationships with symptom severity and other illness metrics (Supplementary Materials).

## Results

### Between-group differences in demographic and clinical measures

There were no significant between-group differences in age, sex, handedness, and IQ (Table 1). SES was significantly higher in OHP versus OBP (*p* < 0.001). SES and IQ were correlated (rho = 0.356, *p* < 0.001); only IQ was thus selected as a covariate in main analyses. For findings using SES as a covariate, see Supplementary Materials.

**Table 1:** Demographic and clinical characteristics of children with bipolar disorder (BD); offspring of bipolar parents (OBP); offspring of comparison parents with non-BD psychopathology (OCP) and healthy offspring of healthy parents (OHP). In OBP and OCP, 23 participants had non-BD diagnoses and 29 were healthy.

	BD^a^ (*n* = 21)	OBP (*n* = 25)	OCP (*n* = 27)	OHP (*n* = 32)	Statistic^b,c^	*p* value^d^
Age at scan (y), mean ± SD^e^	13.89 ± 2.88	13.59 ± 2.49	13.12 ± 2.23	13.19 ± 2.57	H [3] = 2.275	0.517
Female/male	14/7	12/13	12/15	14/18	*X*^2^ [3] = 3.197	0.362
Right/left-handed	21/0	24/1	26/1	30/2	*X*^2^ [3] = 1.355	0.716
Non-BD diagnosis yes/no	19/2	10/15	13/14	32/0	*X*^2^ [2] = 13.447	**0.001**
IQ, mean ± SD	103.10 ± 14	99.76 ± 13.5	101.11 ± 12.1	103.44 ± 13.5	H [3] = 1.963	0.580
Socioeconomic status^f^, mean ± SD	3.48 ± 1.3	2.68 ± 1.3	3.33 ± 1.4	4.09 ± 1.2	H [3] = 15.968	**0.001**
SCARED^g^ - Parent Version, mean ± SD	27.05 ± 16.53	10.36 ± 6.94	11.12 ± 11.25	3.75 ± 5.03	H [3] = 41.391	**0.001**
SCARED—Child Version, mean ± SD	25.57 ± 18.76	14.20 ± 16.07	10.26 ± 13.87	7.81 ± 7.46	H [3] = 15.771	<**0.001**
CALS^h^—Parent Version, mean ± SD	30.42 ± 13.58	8.11 ± 10.39	4.65 ± 6.15	2.34 ± 2.95	H [3] = 45.458	<**0.001**
CALS—Child Version, mean ± SD	28.43 ± 16.2	10.92 ± 10.51	7.85 ± 11.18	3.09 ± 3.93	H [3] = 37.850	<**0.001**
MFQ^i^—Parent Version, mean ± SD	23.47 ± 14.05	5.79 ± 7.75	3.42 ± 3.41	1.90 ± 3.60	H [3] = 44.640	<**0.001**
MFQ—Child Version, mean ± SD	19.95 ± 16.39	8.28 ± 8.32	8.00 ± 11.23	2.94 ± 4.28	H [3] = 23.564	<**0.001**
KMRS^j^	13.86 ± 11.19	2.12 ± 2.98	0.37 ± 0.79	0.13 ± 0.34	H [3] = 35.270	<**0.001**
KDRS^k^	12.19 ± 11.93	2.44 ± 4.43	1.93 ± 3.78	0.29 ± 0.69	H [3] = 29.608	<**0.001**
Offspring Depressive spectrum disorders	4	2	2	0		
Offspring Anxiety spectrum disorders	14	4	10	0		
Offspring Behavioral disorder	7	3	4	0		
Offspring ADHD	11	5	2	0		
Parent (relative) Bipolar disorder	21	BD-I = 16/BD-II = 9	0	0		
Parent Depressive spectrum disorders	4	2	19	0		
Parent Anxiety spectrum disorders	7	21	19	0^l^		
Parent Behavioral disorder	3	18	4	0		
Parent ADHD	1	9	2	0		
Parent Substance use disorders	3	18	11	0		
Parent Axis II disorders	4	12	4	0		

^a^Nine BD had BD I, six had BP II, six had NOS.

^b^The H statistic refers to the Kruskal-Wallis test used to compare groups on non-normally distributed variables.

^c^The chi^2^ statistic refers to the Chi-squared test used to compare groups on relative numbers of participants in specific categories.

^d^*p* values ≤ 0.05 are reported in bold characters.

^e^SD = standard deviation.

^f^SES (1 = 08–19, 2 = 20–29, 3 = 30–39, 4 = 40–54, 5 = 55–66).

^g^SCARED = Screen for Childhood Anxiety and Related Disorders (range, 0–82).

^h^CALS = Child Affect Lability Scale (range, 0–80).

^i^MFQ = Mood and Feelings Questionnaire (Parent version range, 0–68; Child version range, 0–66).

^j^KMRS = K-SADS Mania Rating Scale (range, 0–64).

^k^KDRS = K-SADS Depression Rating Scale (range, 0–61).

^l^Other than mild symptoms of anxiety disorders/PTSD.

As KMRS and KDRS were administered separately from the scan, regression analyses were performed between latency of KSADS ratings and FA in each of the four clusters showing a main effect of group. There was no significant effect of K-SADS latency on FA in each of these four clusters (Supplementary Materials).

### Main effect of group on FA in WM tracts of interest

2.i. There was a significant main effect of group on mean FA across all tracts of interest (*F* [21,262] = 2.58, *p* < 0.001, Wilk’s *Λ* = 0.583, partial *η*^2^ = 0.165).

2.ii. ANCOVAs revealed that there was a significant effect of group on mean FA in bilateral ATR and right CB (Table 2).

**Table 2:** Effects of group on mean FA in each tract of interest.

Tract	*F*	*p* value	FDR corrected *q* value
Forceps minor	0.276	0.842	0.842
Left ATR	**5.692**	**0.001**	**0.004**
Right ATR	**6.580**	**<0.001**	**0.003**
Left CB	2.521	0.062	0.102
Right CB	**4.803**	**0.004**	**0.009**
Left UF	2.393	0.073	0.102
Right UF	1.844	0.144	0.168

*q* values ≤ 0.05 are reported in bold characters.

*ATR* anterior thalamic radiation, *CB* cingulum bundle, *UF* uncinate fasciculus.

**Fig. 1:**
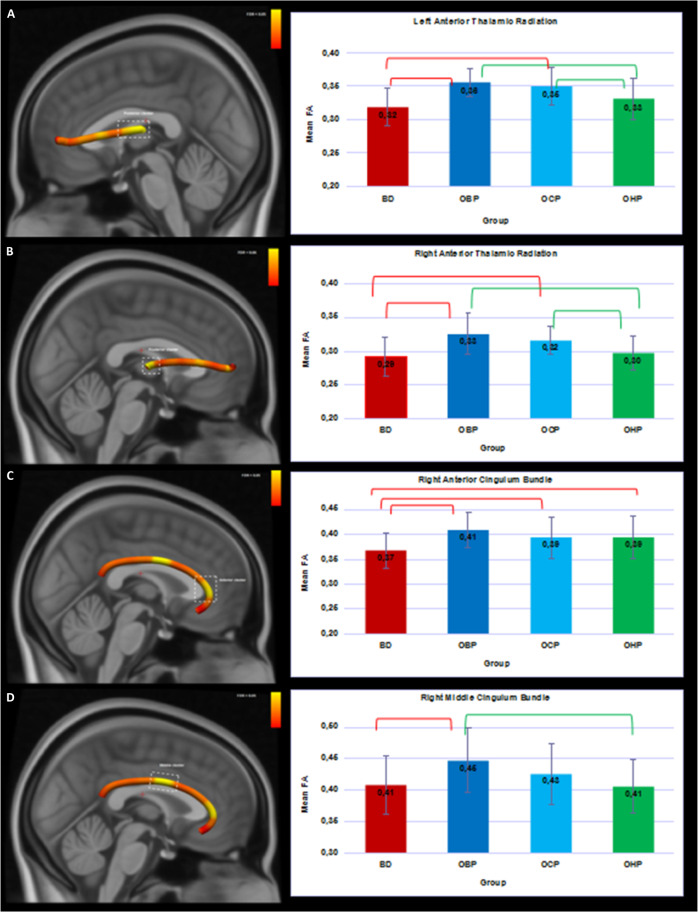
3D visualization of the white matter tracts showing group differences across four groups (BD, OBP, OCP, and OHP). Bar plots represent the mean FA with the main effect of group (FDR-corrected) in four clusters. Red brackets show significant differences in BD versus other groups (*q* < 0.05). Green brackets show significant differences in OHP versus other groups (*q* < 0.05). Error bars represent standard deviations. BD—offspring with BD; OBP—offspring of bipolar parents; OCP—offspring of comparison parents; OHP - healthy offspring of healthy parents. **A** Left Anterior Thalamic Radiation. **B** Right Anterior Thalamic Radiation. **C** Anterior cluster of the Right Cingulum Bundle. **D** Middle cluster of the Right Cingulum Bundle.

2.iii. Tractometry analyses. Four distinct clusters (*q* ≤ 0.05; in >10 consecutive nodes) were identified: in left ATR (*k* = 24 nodes in the proximity of the thalamus; *F* = 6.81; *q* = 0.005), right ATR (*k* = 16 nodes in the proximity of the thalamus; *F* = 6.86; *p* = 0.004), and two clusters in right CB: anterior cluster (*k* = 12 nodes; *F* = 3.97, *p* = 0.01), and middle cluster (*k* = 12 nodes; *F* = 4.00; *p* = 0.01; Fig. 1).

### Between-group differences in clusters

*3.i. Pair-wise comparisons between BD, OBP, OCP, OHP*. BD showed significantly lower FA in the right CB in both clusters than OBP (anterior *q* = 0.003; middle *q* = 0.005), and in the anterior cluster versus OCP (*q* = 0.024), and OHP (*q* = 0.024). BD showed significantly lower FA in the bilateral ATR clusters than OBP and OCP (left: *q* < 0.00, right: *q* < 0.001). OBP showed significantly higher FA in bilateral ATR clusters than OHP(left: *q* = 0.005, right: *q* < 0.001). OBP also showed significantly higher FA in the middle cluster of the right CB than OHP (*q* = 0.003). OCP showed significantly higher FA in bilateral ATR clusters than OHP(left: *q* = 0.017, right: *q* = 0.005; Fig. 1). Effect sizes for all significant pair-wise comparisons were medium to large (Table 3).

**Table 3:** Between-group differences of FA for four groups (BD, OBP, OCP, and OHP)^a^.

**Tract**	**Nodes**^**b**^ **in cluster**	**Comparisons**	**Mean Diff**.^**c**^	**SE**^**d**^	**Cohen’s** ***d***	***p*** **value**	**FDR corrected,** ***q*** **value**^**e**^
Left ATR^f^	**75–98**	BD < OBP	0.038	0.008	1.43	<0.001	<0.001
		BD < OCP	0.034	0.008	1.05	<0.001	<0.001
		BD < OHP	0.015	0.008	0.40	0.056	0.096
		OBP > OCP	0.004	0.007	0.25	0.562	0.613
		OBP > OHP	0.023	0.007	0.91	0.002	0.005
		OCP > OHP	0.019	0.007	0.61	0.008	0.017
Right ATR	**83–98**	BD < OBP	0.034	0.007	1.15	<0.001	<0.001
		BD < OCP	0.028	0.006	0.96	<0.001	<0.001
		BD < OHP	0.010	0.006	0.22	0.130	0.184
		OBP > OCP	0.007	0.006	0.80	0.275	0.314
		OBP > OHP	0.025	0.006	1.01	<0.001	<0.001
		OCP > OHP	0.018	0.006	0.38	0.002	0.005
Right CB^g^	**15–26**	BD < OBP	0.040	0.011	1.14	0.001	0.003
		BD < OCP	0.028	0.011	0.68	0.013	0.024
		BD < OHP	0.027	0.011	0.68	0.013	0.024
		OBP > OCP	0.012	0.010	0.37	0.251	0.301
		OBP > OHP	0.013	0.010	0.36	0.206	0.260
		OCP > OHP	0.001	0.010	0.01	0.936	0.936
Right CB	**50–61**	**BD** < **OBP**	0.041	0.013	0.79	**0.002**	**0.005**
		BD < OCP	0.023	0.013	0.37	0.083	0.125
		BD < OHP	0.002	0.013	0.05	0.860	0.897
		OBP > OCP	0.018	0.012	0.43	0.141	0.188
		**OBP** > **OHP**	0.038	0.012	0.89	**0.001**	**0.003**
		OCP > OHP	0.021	0.011	0.44	0.076	0.122

^a^Group #5 (Converters) had significantly lower FA in right ATR relative to OBP (Table S4).

^b^Each tract divided into 98 nodes.

^c^Mean Diff = Mean Difference.

^d^SE = Standard Error.

^e^*q* values ≤ 0.05 are reported in bold characters.

^f^ATR = anterior thalamic radiation.

^g^CB = cingulum bundle.

Pair-wise *t* tests examining between-group differences using mean RD for each cluster are in Supplementary Table 2. RD was higher in BD than OBP and OCP in most of the clusters. There were no between-group differences in AD.

**Fig. 2:**
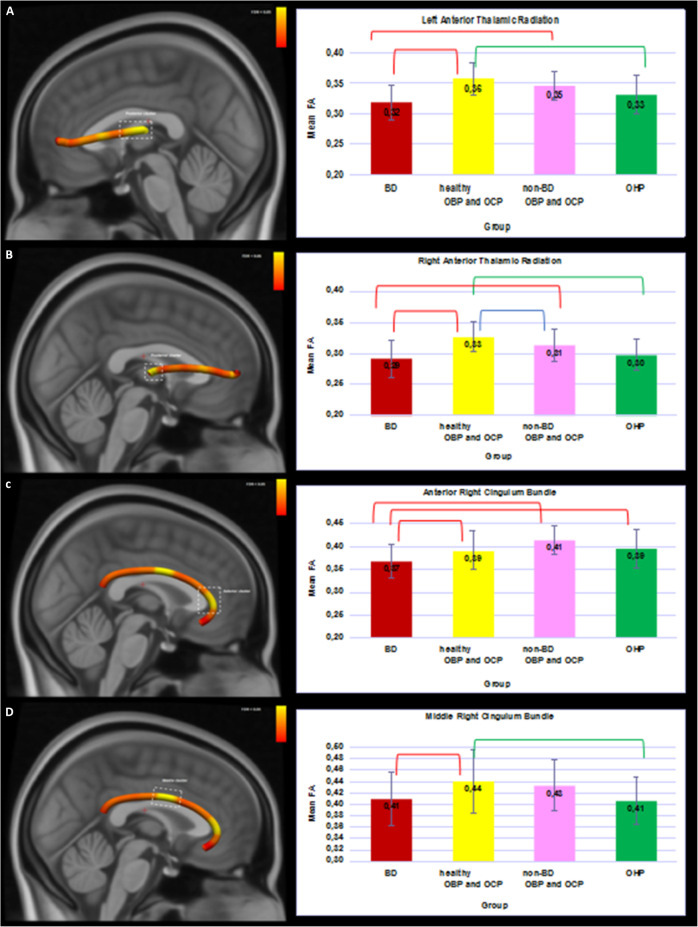
3D visualization of the white matter tracts showing group differences across four groups (BD, healthy OBP and OCP, non-BD OBP and OCP, and OHP). Bar plots represent the mean FA with the main effect of group (FDR-corrected) in 4 clusters. Red brackets show significant differences in BD versus other groups (*q* < 0.05). Green brackets show significant differences in OHP versus other groups (*q* < 0.05). Blue brackets show significant differences between healthy OBP and OCP and non-BD OBP and OCP. Error bars represent standard deviations. BD - offspring with BD; OBP—offspring of bipolar parents; OCP—offspring of comparison parents; OHP—healthy offspring of healthy parents. **A** Left Anterior Thalamic Radiation. **B** Right Anterior Thalamic Radiation. **C** Anterior cluster of the Right Cingulum Bundle. **D** Middle cluster of the Right Cingulum Bundle.

*3.ii. Pair-wise comparisons between, non-BD OBP and OCP (n* = *23), healthy OBP and OCP (n* = *29), and OHP*. In all clusters, healthy OBP and OCP showed higher FA than BD. In bilateral ATR clusters and the middle cluster of the right CB, healthy, but not non-BD, OBP and OCP showed higher FA than OHP. In bilateral ATR clusters and the anterior cluster of the right CB, non-BD OBP and OCP showed higher FA than BD. FA was higher in healthy OBP and OCP versus non-BD OBP and OCP in the right ATR cluster(all *q*s < 0.05; Fig. 2; Supplementary Table 3).

*3.iii. Converters (n* = *5)*. In the cluster in the right ATR (Supplementary Fig. 1), Converters showed lower FA than OBP. There was no difference between Converters and BD (Supplementary Table 4).

*Effects of medication*. Given that 16/21 BD, but very few OBP and OCP, were medicated, exploratory analyses in all BD examined relationships between taking vs. not taking medication and FA in all four clusters showing a main effect of group. These analyses revealed no significant main effect of medication (Supplementary Materials).

## Discussion

We used dMRI and tractometry to identify WM microstructural abnormalities distinguishing BD offspring of BD parents(BD) from non-BD offspring of both BD and non-BD parents (OBP and OCP, respectively) and healthy offspring of healthy parents (OHP). In support of our first hypothesis, BD showed lower FA than OBP and OCP in four clusters in three WM tracts in emotional regulation and reward processing circuitries, including posterior portions of bilateral ATR, and anterior right CB; and in a middle cluster in the right CB versus OHP. Contrary to our second hypothesis, OBP and OCP showed higher bilateral ATR FA than OHP. Furthermore, and in support of our third hypothesis, healthy, but not non-BD, OBP and OCP showed higher middle right CB and bilateral ATR FA than OHP. FA was higher in healthy OBP and OCP versus non-BD OBP and OCP in the right ATR cluster. Exploratory analyses revealed that Converters showed significantly lower FA in this right ATR cluster versus OBP and, at a trend level, versus OCP; and had FA in this cluster at a similar level to BD (slightly higher), rather than OBP and OCP who did not convert.

BD showed significantly lower FA in bilateral ATR and in the anterior cluster of the right CB, and higher RD in most clusters, than both at-risk groups, and significantly lower FA in the anterior right CB cluster than OHP, indicating reduced collinearity and/or lower integrity of fibers, and/or altered myelination [73], in these tracts in BD. FA was also significantly lower in the middle cluster of the right CB in BD versus OBP. Our findings are partially consistent with the existing literature, where youth with BD showed lower FA in the CB relative to youth with a first-degree BD relative [38, 81]. Lower FA in the specific portions of bilateral ATR that we observed might contribute more to emotional dysregulation and aberrant reward processing because of their proximity to subcortical limbic structures involved in emotional regulation and reward processing. Other studies, however, did not report any differences in FA in the ATR in BD versus healthy OBP [82], in BD versus their healthy first-degree relatives [39], and in BD versus youth with a first-degree BD relative [32, 38]. Additionally, previous studies [32, 33, 35–37, 83, 84] and a recent meta-analysis [30] reported significantly lower FA in BD than healthy controls in several tracts. Younger age of BD onset and longer BD duration was associated with higher FA in tracts showing between-group differences in FA (Supplementary Materials). These findings might reflect the fact that almost all BD were taking medication (mean duration = 2.48 years), which might have had an ameliorative effect on FA [85, 86].

Contrary to our second hypothesis, bilateral ATR FA was higher in OBP and OCP than OHP, and FA in the middle cluster of the right CB was higher in OBP than OHP. Furthermore, in bilateral ATR clusters and the middle cluster of the right CB, healthy, but not non-BD, OBP and OCP showed higher FA than OHP. Only one previous study compared OBP, OCP, and OHP [25]. Here, lower FA in OBP and OCP versus OHP was in left-sided tracts(including the CB, inferior longitudinal fasciculus, and forceps minor), but higher FA in OBP and OCP versus OHP was observed in right-sided tracts(including UF and inferior longitudinal fasciculus), paralleling our findings of higher FA in predominantly right-sided tracts in OBP and OCP versus OHP. These two studies employed different methodologies [56, 87, 88], and the previous study included a larger number of OBP, OCP, and OHP: four OBP, five OCP, two OHP were excluded from the current study due to poor reconstruction using TractSeg. In the previous study, the four OBP and one OCP Converters were excluded. In the present study, Converters were included, and OBP and OCP had not converted to BD despite a mean follow-up period of 5.2 years post scan.

The ATR and CB support executive [89, 90] function and emotional regulation [91–93]. Thus, our findings of elevated FA in bilateral ATR in OBP and OCP, and in the right CB in OBP, and the fact that this pattern of elevated FA was evident only in healthy OBP and OCP, suggest a compensatory rearrangement of the fibers in bilateral ATR to overcome familial predisposition to psychiatric disorders in general, and possibly in the right CB to overcome familial predisposition to BD [25, 94]. Indeed, elevated FA has been proposed as a compensatory mechanism in neurodegenerative diseases [95], and elevated FA in motor tracts, a compensatory reorganization of WM fibers in Parkinson’s disease, possibly reflecting adaptive or extended neuroplasticity [96].

Non-BD OBP and OCP had significantly lower FA in the right ATR cluster than healthy OBP and OCP, and did not show significant differences in FA in this cluster than OHP. Furthermore, despite a small sample size, our exploratory analyses revealed that Converters also had lower FA than OBP in the right ATR cluster. Alongside our findings above showing that elevated FA in right(and left) ATR might protect against psychiatric disorders, these findings suggest that absence of elevated right ATR FA might result in lack of protection against future BD in OBP and OCP. By contrast, low anterior right CB FA might represent a neural correlate of having BD, given that this was the only tract in which BD showed significantly lower FA than OHP. Together, these data highlight a potential role of the right hemisphere in protective mechanisms against behaviors related to dysregulation and BD, depending upon the magnitude of structural, and potentially, functional measures in circuitries supporting emotional regulation and reward processing. Indeed, greater activity in the right prefrontal cortex has been shown to be associated with greater risk aversion [97]. Our findings also parallel reports highlighting how diffusivity measures in the CB might predict resilience and impulsiveness in BD at-risk youth [98], and provide potential neural targets for early interventions in at-risk youth [99, 100].

Certain limitations should be considered. Although effect sizes in the present study are medium to large, future studies with larger samples sizes should aim to replicate these findings, especially abnormally elevated FA in OBP and OCP in bilateral ATR. Our subgroup of converters was small, although this sample, which was ~10% of the size of combined OBP and OCP, is similar to reported conversion rates in OBP and OCP [9–11]. Converters were ~2 years older than other groups, indicating the need for longer follow-up in order to include a larger number of Converters in future studies. While most of our mood-related measures were assessed on the day of the scan, the KMRS and KDRS were assessed on average within two months before or after the scan day. Regression analyses examining relationships between the latency of the KMRS and KDRS ratings and FA in clusters showing a main effect of group revealed no significant effects, however (Supplementary Materials). The novel tractometry approach used to examine WM tracts differed from that used in our previous study [23], with differences, although some similarities, in findings between the two studies. The majority of BD was medicated, but only three OCP and two OB, were medicated. Exploratory analyses did not reveal any effect of taking medication on FA in any of the four clusters in BD, however (Supplementary Materials).

This is the first dMRI study comparing offspring with BD and offspring at risk for BD and/or other psychopathology with prospective follow-up, allowing comparison of those who converted to BD with those who did not do so. We highlight a potential role of dMRI in identifying objective biomarkers that might reflect underlying neural mechanisms that predispose to versus protecting against future BD and non-BD psychopathology, to help identify youth most at risk of future BD.

### Funding and disclosure

The present study was supported by the National Institute of Mental Health grant R01 MH060952-16 (MPIs: Boris Birmaher and Mary L. Phillips), and by the Pittsburgh Foundation (Mary L. Phillips). These funding institutions were not involved in the design or conduct of the study, the collection, management, analysis, or interpretation of the data, or the preparation, review, or approval of the manuscript.

Renata Rozovsky, Amelia Versace, Michele Bertocci, Cecile D. Ladouceur, Mary L. Phillips and Lisa K. Bonar, Kelly Monk, Halimah Abdul-Waalee, and Genna Bebko do not have any financial disclosures. Jay Fournier receives royalties from Guilford Press and has served as a paid consultant to Happify.com. Danella Hafeman receives funding from the Brain and Behavior Research Foundation. Boris Birmaher has or will receive royalties for publications from: Random House, Inc. (New Hope for Children and Teens with Bipolar Disorder), Lippincott Williams & Wilkins (Treating Child and Adolescent Depression), Wolters Kluwer. Tina Goldstein receives royalties from Guilford Press. Dara Sakolsky received an honorarium from Northwell Health in 2018.

## Supplementary information

Differentiating white matter measures that protect against vs. predispose to Bipolar Disorder and other psychopathology in at-risk youth
