# Supplementary material for: Differentiating white matter measures that protect against vs. predispose to bipolar disorder and other psychopathology in at-risk youth
Source: Neuropsychopharmacology. 2021 Jul 20;46(12):2207–16. doi: 10.1038/s41386-021-01088-1 (PMC8505429; doi:10.1038/s41386-021-01088-1)

**Differentiating white matter measures that protect against vs. predispose to Bipolar Disorder and other psychopathology in at-risk youth**

Renata Rozovsky^1^, Amelia Versace^1,2^, Lisa K. Bonar^1^, Michele Bertocci^1^, Cecile D. Ladouceur^1^, Jay Fournier^1^, Kelly Monk^1^, Halimah Abdul-waalee^1^, Genna Bebko^1^, Danella Hafeman^1^, Dara Sakolsky^1^, Tina Goldstein^1^, Boris Birmaher^1^, Mary L. Phillips^1^.

1. Department of Psychiatry, Western Psychiatric Institute and Clinic, University of Pittsburgh Medical Center, University of Pittsburgh, Pittsburgh, PA, USA.
2. Magnetic Resonance Research Center, Department of Radiology, University of Pittsburgh Medical Center, University of Pittsburgh, Pittsburgh, Pennsylvania.

**Corresponding author**

Renata Rozovsky (PhD)

302 Loeffler Building

121 Meyran Ave.

Pittsburgh Pa 15213

Phone: 412-383-8206, fax: 412-383-8336

e-mail: rozovskyr@upmc.edu

**Supplemental Materials**

**Exclusion criteria**

Exclusion criteria included: a history of serious medical illness, head injury, or neurological disorder; IQ <70, as assessed by the Wechsler Abbreviated Scale of Intelligence(1); diagnosis of BD (except for the BD group), autism, or schizophrenia; MRI contraindication (e.g., pregnancy, metal in the body). For OHP, additional exclusion criteria included any current and/or lifetime history of any Axis-I and Axis-II disorder and family history (1^st^ degree) of psychiatric illness other than mild anxiety disorders/PTSD. Eleven participants were excluded after dMRI data analysis due to movements and/or poor reconstruction of the data.

**Clinical assessment**

Psychiatric diagnoses were confirmed by a licensed psychiatrist or psychologist before the neuroimaging assessment using the Kiddie Schedule for Affective Disorders and Schizophrenia for School-Age Children (K-SADS) - Present and Lifetime Version(2) for offspring (inter-rater reliability for K-SADS is 0.80), and the Structural Clinical Interview for DSM-IV(3) and Family History Screen(4) for parents. All biological parents (who participated in the study), including parents of healthy offspring (OHP), at-risk offspring (OBP and OCP) and offspring with a BD diagnosis (BD) completed the Structured Clinical Interview for DSM-IV (SCID) interview at the beginning of the study.  However, inclusion criteria for the BD group was to have a 1^st^ or 2^nd^ degree relative with BD. If the biological parent taking part in the study was not the BD relative, a Family History interview was completed to collect information on 1^st^  or 2^nd^ degree relatives with a BD diagnosis.

Offspring assessments included the Screen for Child Anxiety Related Disorders (SCARED)(5,6), Children’s Affective Lability Sale (CALS)(7), Mood and Feelings Questionnaire (MFQ)(8), to assess depressive symptoms, K-SADS Mania Rating Scale (KMRS)(9), and K-SADS Depression Rating Scale (KDRS)(2). Parent- and child-reported SCARED, CALS, and MFQ were administered on the scan day. The K-SADS, KMRS and KDRS assessments, based on both parent and child information, were administered every two years after intake in all offspring groups and at, on average, two months after the scan. All participants also completed medication forms that documented psychotropic medications used at the time of the scan. Sixteen BD participants were taking psychotropic medications (stimulants, antipsychotics, antidepressants, benzodiazepines, mood stabilizers). Two OBP participants were taking psychotropic medications for non-BD diagnoses (one was taking a stimulant, and one a non-stimulant). Three OCP were taking psychotropic medications for non-BD diagnoses (two participants were taking antidepressants and one was taking stimulants). Handedness was assessed using the Annett Behavioral Handedness Index (10). Interviewers also evaluated socioeconomic status(SES)(11).

**Diffusion imaging acquisition**

Images were acquired on a 3T Siemens Trio and Prisma systems at the Magnetic Resonance Research Center, University of Pittsburgh Medical Center Health System, USA. A standard body coil was used for RF transmission, while the MR signal was acquired with a Siemens 12 channels receiver only coil. The gradient coil set achieves 40mT/m maximum amplitude and a slew rate of 200mT/m/sec. The combination of the array coils and multiple RF receivers enables the use of parallel imaging techniques, reducing the total scan time (up to 40%) and therefore the EPI-based distortions (12). Restraining foam pads were utilized for minimizing head motion. Diffusion-weighted images were acquired using single-shot, spin-echo planar imaging (EPI), Siemens product sequence (double spin echo, bipolar gradients). This sequence markedly ameliorates the eddy current distortions of diffusion-weighted imaging (13). The anterior to posterior (or posterior to anterior) phase encode direction results in better quality data in terms of ghosts and distortions relative to left to right (L>>R) direction (14). To avoid the compression of voxels near tissue-air interfaces such as in the frontal lobe that would have resulted from an A>>P direction, we adopted a posterior to anterior (P>>A) phase-encode direction. Sixty-one optimized non-collinear diffusion gradient directions (b=1000 s/mm2) were acquired along with seven b0 images (interleaved every 9 non-collinear images). Planes were parallel to the AC–PC line (TR=8400ms; TE=90ms; bandwidth 1860[Hz/Px]; flip angle=90; field-of-view [FOV] =256x256; sixty-four 2 mm-thick slices; no gaps; matrix size=128x128; EPI factor=128; no acceleration factor was used; acquisition duration: 9'56"). Anatomical images covering the entire brain were acquired using an axial 3D-MPRAGE sequence, parallel to the AC–PC line (TE/TI/TR=3.29ms/900ms/2200ms, flip angle=9, isotropic 1mm3 voxel, 192 axial slices, matrix size=256x192; acquisition time: 7'02").

**Diffusion imaging data analysis**

Diffusion-weighted images were transferred to a Unix-based workstation. Distortions due to eddy currents and head motion were reduced using *eddy_correct* (15) within the Functional Magnetic Resonance Imaging of the Brain Software Library (FSL; http://www.fmrib.ox.ac.uk/fsl). The gradient vectors were rotated accordingly to the spatial transformations applied to their corresponding volumes using *fdt_rotate_bvecs* within the same package (FSL; http://www.fmrib.ox.ac.uk/fsl). Skull and non-brain voxels were stripped using the brain extraction tool (bet) (16) within FSL (17). Tracts were segmented in native space using TractSeg (18). TractSeg employs a convolutional neural network-based approach (i.e., Tract Orientation Mapping) to automatically segment 50 major WM tracts using the field of fiber orientation distribution function (fODF), as implemented in mrtrix-3 (19). Tract Orientation Mapping facilitates bundle-specific tractography based on a learned mapping from the original fODF peaks to a list of tract orientation maps (TOM) (20). Each TOM represents one of the known tracts with each voxel containing no more than one orientation vector. TractSeg allows for a point-wise tractographic approach (i.e., tractometry). Thus, diffusion imaging measures (e.g., FA, RD, AD) were extracted in *98* nodes along each tract (point-wise values) and averaged across the entire tract (mean values) and exported into SPSS (version 26).

**Supplemental analyses**

*Relationships between symptom severity and FA findings.* Given non-parametric distributions of these variables, *Spearman’s correlations were used to explore the relationships between FA in all four clusters and* anxiety as measured by the SCARED; mood lability as measured by the CALS; depression as measured by the MFQ and KDRS; and mania as measured by the KMRS.

*Relationships between the age of BD onset, illness duration, and FA findings.* Given non parametric distributions of these measures, Spearman’s correlations were used to explore these relationships in BD.

*Effects of non-BD disorders (yes/no)* *on FA findings.* Four separate MANOVAs were used to explore the effect of either Depressive Disorders, Anxiety Disorders, Behavioral Disorders, or ADHD as an additional covariate on mean FA in cluster(s) showing a main effect of group across BD, OBP, and OCP.

*Effects of age.* To assess effects of age, four Spearman’s correlation analyses (one for each cluster) were performed using age as an independent variable, and FA in the relevant cluster as the dependent variable.

*Effects of medication.* Four parallel ANOVAs (one for each cluster) were performed in BD using psychotropic medication (taking vs. not taking) as an independent variable.

**Results**

**Between-group differences in demographic and clinical measures.**

BD had significantly more anxiety as measured by the SCARED relative to other groups (parent version BD vs OBP, OCP, OHP q<0.05; child version BD vs OCP, OHP q<0.05). BD also had significantly higher mood lability as measured by the CALS (both parent and child versions BD vs OBP, OCP, OHP q<0.05), and depression symptoms as measured by the MFQ (both parent and child versions BD vs OBP, OCP, OHP q<0.05). BD also had significantly higher mania as measured by the KMRS (OBP, OCP, OHP q<0.05) and depression as measured by KDRS (OBP, OCP, OHP q<0.05). OBP had significantly higher anxiety as measured by the SCARED (parent version q<0.05,), and affective lability as measured by the CALS (both parent and child versions q<0.05), depression symptoms as measured by the MFQ (both parent and child versions q<0.05), mania as measured by the KMRS (q<0.05), and depression as measured by KDRS (q<0.05) than OHP. OCP had significantly higher anxiety as measured by SCARED relative to OHP (parent version q<0.05). (Supplemental Table 1).

**Relationships between symptom severity and FA findings**

There was a significant negative correlation between FA in all clusters and anxiety, mood lability, affective symptoms, depression, and mania across BD, OBP, and OCP. Specifically, there was a significant negative correlation across BD, OBP, and OCP between FA in the left ATR cluster and: anxiety, as measured by the SCARED (parent and child versions, rho=-0.357 and -0.445 respectively, q<0.05); mood lability, as measured by the CALS (parent version, rho=-0.347, p<0.05, child version, rho=-0.550, q<0.001); mania, as measured by the KMRS (rho=-0.409, q<0.001); affective symptoms, as measured by the MFQ (child version, rho=-0.385, q<0.05); and depression as measured by the KDRS (rho=-0.374, q<0.05). There were negative correlations between FA in the right ATR cluster and: anxiety, as measured by the SCARED (parent and child versions, rho=-0.350 and -0.367 respectively, q<0.05); mood lability, as measured by the CALS (child version, rho=-0.446, q<0.05); and mania, as measured by the KMRS (rho=-0.387, q<0.05). There was also a significant negative correlation across BD, OBP, and OCP between FA in the anterior cluster of the right CB and mood lability, as measured by the CALS (parent version, rho=-0.346, q<0.05); and affective symptoms, as measured by the MFQ (parent version, rho=-0.339, q<0.05). There was a similar negative correlation between FA in the middle cluster of the right CB and anxiety, as measured by the SCARED (child version, rho=-0.321, q<0.05).

When analyses were performed in each group separately, there was a significant negative correlation in BD between FA in the left ATR cluster and: anxiety, as measured by the SCARED (parent version, rho=-0.699, q<0.05); mood lability, as measured by the CALS (child version, rho=--632, q<0.05); mania, as measured by the KMRS (rho=-0.624, q<0.05). There were no significant correlations in OBP and OCP.

**Relationships among age of onset of BD, illness duration, and FA findings**

There was a negative correlation between age of BD onset and mean FA in the middle cluster of the right CB (rho=-0.572, q=0.049). There was a positive correlation between illness duration and mean FA in the left ATR cluster; however, it did not survive correction for multiple comparisons (rho=0.438, p=0.047,uncorrected; Supplemental Table 6).

**Non-BD disorders**

Across BD, OBP and OCP, MANOVAs did not reveal any effect of Depressive Disorders, Anxiety, Behavioral Disorders, and ADHD on any FA cluster showing a main effect of group: ADHD(F(4,62)=1.81, Willks' Λ = 0.895, p=0.137, partial η2=0.105), Depressive disorders (F(4,62)=0.59, Willks' Λ = 0.963, p=0.672, partial η2=0.037); Behavioral disorders (F(4,62)=1.534, Willks' Λ = 0.921, p=0.266, partial η2=0.079); Anxiety disorders (F(4,62)=1.55, Willks' Λ = 0.909, p=0.199, partial η2=0.091). The different comorbid diagnostic groups (e.g, depressive disorders) were likely very heterogenous in nature, which is a possible reason why there were no significant differences in FA among these different comorbid disorders.

**Effects of age**

Spearman’s correlation analyses revealed positive correlations between age and FA in all four clusters (left ATR: rho=0.280, q<0.05; right ATR: rho=0.434, q<0.001; anterior cluster of the right CB: rho=0.263, q<0.05; middle cluster of the right CB: rho=0.381, q<0.001).

**Effects of medication**

In BD, ANOVAs (one for each cluster) did not reveal any significant effects of medication on FA: left ATR q=0.214; right ATR q=0.573; anterior cluster of the right CB q=0.214; middle cluster of the right CB q=0.295.

**Exploratory analysis of effects of offspring diagnoses and parental diagnoses**

Multivariate Analysis of Variance (MANOVA) was first performed to identify the main effect of Offspring diagnosis (3 levels: BD, non-BD, healthy) and Parental diagnosis (3 levels: BD, non-BD, healthy) on mean FA in the seven tracts of interest: the forceps minor, and three bilateral tracts: ATR, CB, and UF. Offspring diagnosis and Parental diagnosis were the independent variables; age, sex, IQ, and handedness were covariates; and mean FA in the 7 tracts was the multiple dependent variable. There was a significant main effect of Offspring diagnosis on mean FA across all tracts of interest (F(14,180)=2.93,p<0.001, Wilk's Λ=0.664, partial η2=0.185). Main effect of Parental diagnosis was not significant (F(14,180)=1.62,p=0.078, Wilk's Λ=0.789, partial η2=0.112).

Univariate analyses (seven parallel ANCOVAs; one for each tract of interest) were performed to identify which tract contributed to the main effect of Offspring diagnosis across all tracts of interest. Offspring diagnosis and covariates were the independent variables, and mean FA in each tract was the dependent variable. ANCOVAs revealed that there was a significant effect of Offspring diagnosis on mean FA in Right ATR (p = 0.008, q = 0.036), Left ATR (p = 0.002, q = 0.015) and Right CB (p = 0.002, q = 0.015).

Pair-wise comparisons between Offspring diagnosis (3 levels: BD, non-BD, healthy) and Parental diagnosis (3 levels: BD, non-BD, healthy) showed that the main effect of group of FA in Right ATR was driven by: Offspring with non-BD > Offspring with BD (p = 0.006, q = 0.020), and by healthy Offspring > Offspring with BD (p = 0.004, q = 0.017). In Left ATR, the main effect of group on FA was driven by: Offspring with non-BD > Offspring with BD (p = 0.001, q = 0.010), and healthy Offspring > Offspring with BD (p = 0.002, q = 0.010). In Right CB, the main effect of group was driven by: Offspring with non-BD > Offspring with BD (p = 0.001, q = 0.010) and healthy Offspring > Offspring with BD (p = 0.002, q = 0.010).

These additional findings parallel our main findings indicating lower FA in the right CB in BD versus other groups, and in OHP versus OBP, lower bilateral ATR FA in BD versus OBP and OCP, and higher FA in bilateral ATR in OBP and OCP than both BD and OHP. These additional analyses do not allow any inferences to be made about the impact of familial history of BD on FA in these tracts, however, as our sample size was not powered to examine any interactions between offspring and parental diagnoses on FA.

**Sensitivity analysis**

Two OCP participants had no parents with a non-BD diagnosis, but they had themselves an Anxiety Disorder or Depressive Disorder. A sensitivity analysis excluding these two OCP participants was conducted. This did not affect the main findings. The following is a summary of the differences identified by the sensitivity analysis regarding main findings:

- 7 consecutively significant segments in the anterior cluster in the right CB instead of 10.
- FA differences in the middle cluster of the right CB (non-BD OBP and OCP > OHP and non-BD OBP and OCP > BD)
- FA correlation with SCARED score (parent version) in the anterior cluster of the right CB.

**Analyses using SES as a covariate** **instead of IQ**

Parallel ANCOVAs for each tract/cluster using SES instead of IQ did not reveal any changes in our main findings: findings regarding the main effect of group on FA in 7 tracts of interest were unchanged (Table S7).

**Regression analysis examining the effect of latency between K-SADS assessment and FA in all four clusters**

Linear regression analyses examining the main effect of latency between K-SADS assessment and scan on FA in the four clusters showing a main effect of group did not reveal any significant relationships: Left ATR (F=0.058, q = 0.810); Right ATR (F=3.894, q = 0.204 ); anterior cluster of the right CB (F=0.100, q = 0.810 ); middle cluster of the right CB (F=0.920, q = 0.680 ).

**Supplemental discussion**

Although there was insufficient power to examine interactions between age and group, we performed analyses assessing effects of age as a covariate in the main model, and additional analyses of relationships between age and FA. These analyses revealed positive correlations between age and FA in all four clusters. The effects of age on FA and the directionality of these effects are consistent with the extant literature showing that in normal development FA increases during puberty across a large number of white matter tracts(21–27), although the effects of age on these FA trajectories in BD youth need to be examined in larger samples(28,29).

**Supplemental References**

1. Wechsler, D. Wechsler abbreviated scale of intelligence. San Antonio, TX: The Psychological Corporation; 1999.

2. Kaufman J, Birmaher B, Brent D, Rao U, Flynn C, Moreci P, et al. Schedule for Affective Disorders and Schizophrenia for School-Age Children-Present and Lifetime Version (K-SADS-PL): initial reliability and validity data. J Am Acad Child Adolesc Psychiatry. 1997 Jul;36(7):980–8.

3. First MB, Gibbon ML, Spitzer RL, Williams JBW, Benjamin L. Structured Clinical Interview for DSM-IV Axis II Personality Disorders (SCID-II). Washington, DC: American Psychiatric Press; 1997.

4. Weissman MM, Wickramaratne P, Adams P, Wolk S, Verdeli H, Olfson M. Brief screening for family psychiatric history: the family history screen. Arch Gen Psychiatry. 2000 Jul;57(7):675–82.

5. Birmaher B, Brent DA, Chiappetta L, Bridge J, Monga S, Baugher M. Psychometric properties of the Screen for Child Anxiety Related Emotional Disorders (SCARED): a replication study. J Am Acad Child Adolesc Psychiatry. 1999 Oct;38(10):1230–6.

6. Birmaher B, Khetarpal S, Brent D, Cully M, Balach L, Kaufman J, et al. The Screen for Child Anxiety Related Emotional Disorders (SCARED): scale construction and psychometric characteristics. J Am Acad Child Adolesc Psychiatry. 1997 Apr;36(4):545–53.

7. Gerson AC, Gerring JP, Freund L, Joshi PT, Capozzoli J, Brady K, et al. The Children’s Affective Lability Scale: a psychometric evaluation of reliability. Psychiatry Res. 1996 Dec 20;65(3):189–98.

8. Sund AM, Larsson B, Wichstrom L. Depressive symptoms among young Norwegian adolescents as measured by the Mood and Feelings Questionnaire (MFQ). Eur Child Adolesc Psychiatry. 2001 Dec;10(4):222–9.

9. Axelson D, Birmaher BJ, Brent D, Wassick S, Hoover C, Bridge J, et al. A preliminary study of the Kiddie Schedule for Affective Disorders and Schizophrenia for School-Age Children mania rating scale for children and adolescents. J Child Adolesc Psychopharmacol. 2003;13(4):463–70.

10. Annett M. A classification of hand preference by association analysis. Br J Psychol. 1970 Aug;61(3):303–21.

11. Hollingshead, A. Four-factor index of social status. New Haven, CT: Yale University; 1975.

12. Bammer R, Auer M, Keeling SL, Augustin M, Stables LA, Prokesch RW, et al. Diffusion tensor imaging using single-shot SENSE-EPI. Magnetic Resonance in Medicine. 2002;48(1):128–36.

13. Finsterbusch J. Double-spin-echo diffusion weighting with a modified eddy current adjustment. Magn Reson Imaging. 2010 Apr;28(3):434–40.

14. Poustchi-Amin M, Mirowitz SA, Brown JJ, McKinstry RC, Li T. Principles and applications of echo-planar imaging: a review for the general radiologist. Radiographics. 2001 Jun;21(3):767–79.

15. Jenkinson M, Bannister P, Brady M, Smith S. Improved optimization for the robust and accurate linear registration and motion correction of brain images. Neuroimage. 2002 Oct;17(2):825–41.

16. Smith SM. Fast robust automated brain extraction. Hum Brain Mapp. 2002 Nov;17(3):143–55.

17. Smith SM, Jenkinson M, Woolrich MW, Beckmann CF, Behrens TEJ, Johansen-Berg H, et al. Advances in functional and structural MR image analysis and implementation as FSL. Neuroimage. 2004;23 Suppl 1:S208-219.

18. Wasserthal J, Neher P, Maier-Hein KH. TractSeg - Fast and accurate white matter tract segmentation. NeuroImage. 2018 Dec 1;183:239–53.

19. Tournier J-D, Smith R, Raffelt D, Tabbara R, Dhollander T, Pietsch M, et al. MRtrix3: A fast, flexible and open software framework for medical image processing and visualisation. NeuroImage. 2019 Nov 15;202:116137.

20. Wasserthal J, Neher PF, Maier-Hein KH. Tract orientation mapping for bundle-specific tractography. arXiv:180605580 [cs] [Internet]. 2018 Jun 14 [cited 2020 Oct 28]; Available from: http://arxiv.org/abs/1806.05580

21. Genc S, Malpas CB, Gulenc A, Sciberras E, Efron D, Silk TJ, et al. Longitudinal patterns of white matter fibre density and morphology in children are associated with age and pubertal stage. Dev Cogn Neurosci. 2020 Oct;45:100853.

22. Bonekamp D, Nagae LM, Degaonkar M, Matson M, Abdalla WMA, Barker PB, et al. Diffusion Tensor Imaging in Children and Adolescents: Reproducibility, Hemispheric, and Age-Related Differences. Neuroimage. 2007 Jan 15;34(2):733–42.

23. Schmithorst VJ, Wilke M, Dardzinski BJ, Holland SK. Correlation of White Matter Diffusivity and Anisotropy with Age during Childhood and Adolescence: A Cross-sectional Diffusion-Tensor MR Imaging Study. Radiology. 2002 Jan;222(1):212–8.

24. Aa O, S A, Jp L, C R, Ku S, N S, et al. Development of short-range white matter in healthy children and adolescents. Hum Brain Mapp. 2018;39:204–17.

25. Tamnes CK, Roalf DR, Goddings A-L, Lebel C. Diffusion MRI of white matter microstructure development in childhood and adolescence: Methods, challenges and progress. Developmental Cognitive Neuroscience. 2018 Oct 1;33:161–75.

26. Brouwer RM, Mandl RCW, Schnack HG, van Soelen ILC, van Baal GC, Peper JS, et al. White Matter Development in Early Puberty: A Longitudinal Volumetric and Diffusion Tensor Imaging Twin Study. PLoS One [Internet]. 2012 Apr 13 [cited 2021 May 25];7(4). Available from: https://www.ncbi.nlm.nih.gov/pmc/articles/PMC3326005/

27. Barnea-Goraly N, Menon V, Eckert M, Tamm L, Bammer R, Karchemskiy A, et al. White matter development during childhood and adolescence: a cross-sectional diffusion tensor imaging study. Cereb Cortex. 2005 Dec;15(12):1848–54.

28. Weathers J, Lippard ETC, Spencer L, Pittman B, Wang F, Blumberg HP. Longitudinal Diffusion Tensor Imaging Study of Adolescents and Young Adults With Bipolar Disorder. J Am Acad Child Adolesc Psychiatry. 2018;57(2):111–7.

29. Serafini G, Pompili M, Borgwardt S, Houenou J, Geoffroy PA, Jardri R, et al. Brain changes in early-onset bipolar and unipolar depressive disorders: a systematic review in children and adolescents. Eur Child Adolesc Psychiatry. 2014 Nov 1;23(11):1023–41.

***Table S1. Clinical between-group differences for 4 groups (BD, OBP, OCP, and OHP).***

| **Measure** | **Comparisons** | **Test statistic** | **SE^a^** | **p-value** | **FDR**  **corrected^b^ q-value** |
| --- | --- | --- | --- | --- | --- |
| SCARED^c^ – parent version | **BD > OBP** | 25.983 | 8.911 | 0.004 | **0.009** |
|  | **BD > OCP** | 29.316 | 8.832 | 0.001 | **0.003** |
|  | **BD > OHP** | 53.846 | 8.454 | 0.004 | **0.009** |
|  | OBP > OCP | 3.333 | 8.432 | 0.693 | 0.357 |
|  | **OBP > OHP** | 27.863 | 8.035 | 0.001 | **0.003** |
|  | **OCP > OHP** | 24.530 | 7.948 | 0.002 | **0.005** |
| SCARED – child version | BD > OBP | 18.580 | 9.002 | 0.039 | 0.055 |
|  | **BD > OCP** | 30.185 | 8.848 | 0.001 | **0.003** |
|  | **BD > OHP** | 30.563 | 8.540 | <0.001 | **<0.001** |
|  | OBP > OCP | 11.605 | 8.441 | 0.169 | 0.208 |
|  | OBP > OHP | 11.983 | 8.117 | 0.140 | 0.177 |
|  | OCP > OHP | 0.377 | 7.947 | 0.962 | 0.962 |
| CALS^d^ - parent version | **BD > OBP** | 36.041 | 8.862 | <0.001 | **<0.001** |
|  | **BD > OCP** | 45.439 | 8.784 | <0.001 | **<0.001** |
|  | **BD > OHP** | 54.787 | 8.408 | <0.001 | **<0.001** |
|  | OBP > OCP | 9.398 | 8.386 | 0.262 | 0.299 |
|  | **OBP > OHP** | 18.746 | 7.992 | 0.019 | **0.030** |
|  | OCP > OHP | 9.349 | 7.905 | 0.237 | 0.277 |
| CALS - child version | **BD > OBP** | 29.283 | 8.968 | 0.001 | **0.003** |
|  | **BD > OCP** | 39.272 | 8.815 | <0.001 | **<0.001** |
|  | **BD > OHP** | 51.096 | 8.508 | <0.001 | **<0.001** |
|  | OBP > OCP | 9.990 | 8.409 | 0.235 | 0.277 |
|  | **OBP > OHP** | 21.813 | 8.087 | 0.007 | **0.013** |
|  | OCP > OHP | 11.823 | 7.917 | 0.135 | 0.175 |
| MFQ^e^ - parent version | **BD > OBP** | 36.819 | 8.805 | <0.001 | **<0.001** |
|  | **BD > OCP** | 38.869 | 8.727 | <0.001 | **<0.001** |
|  | **BD > OHP** | 55.416 | 8.353 | <0.001 | **<0.001** |
|  | OBP > OCP | 2.050 | 8.332 | 0.806 | 0.823 |
|  | **OBP > OHP** | 18.597 | 7.940 | 0.019 | **0.030** |
|  | OCP > OHP | 16.547 | 7.853 | 0.035 | 0.051 |
| MFQ – child version | **BD > OBP** | 19.496 | 8.891 | 0.028 | **0.043** |
|  | **BD > OCP** | 27.661 | 8.740 | 0.002 | **0.005** |
|  | **BD > OHP** | 40.101 | 8.436 | <0.001 | **<0.001** |
|  | OBP < OCP | 8.165 | 8.337 | 0.327 | 1.000 |
|  | **OBP > OHP** | 20.605 | 8.018 | 0.010 | **0.017** |
|  | OCP > OHP | 12.440 | 7.849 | 0.113 | 0.151 |
| KMRS^f^ | **BD > OBP** | 20.602 | 7.698 | 0.007 | **0.013** |
|  | **BD > OCP** | 35.817 | 7.567 | <0.001 | **<0.001** |
|  | **BD > OHP** | 40.294 | 7.350 | <0.001 | **<0.001** |
|  | OBP > OCP | 15.216 | 7.218 | 0.035 | 0.051 |
|  | **OBP > OHP** | 19.692 | 6.991 | 0.005 | **0.010** |
|  | OCP > OHP | 4.477 | 6.846 | 0.513 | 0.547 |
| KDRS^g^ | **BD > OBP** | 21.757 | 8.189 | 0.008 | **0.014** |
|  | **BD > OCP** | 29.561 | 8.049 | <0.001 | **<0.001** |
|  | **BD > OHP** | 41.776 | 7.819 | <0.001 | **<0.001** |
|  | OBP > OCP | 7.804 | 7.679 | 0.309 | 0.345 |
|  | **OBP > OHP** | 20.019 | 7.437 | 0.007 | 0.013 |
|  | OCP > OHP | 12.216 | 7.283 | 0.093 | 0.128 |
| ^a^ SE = Standard Error  ^b^ q-values ≤ 0.05 are reported in bold characters  ^c^SCARED = Screen for Childhood Anxiety and Related Disorders (range, 0-82)  ^d^CALS = Child Affect Lability Scale (range, 0-80)  ^e^MFQ = Mood and Feelings Questionnaire (range, 0-68)  ^f^KMRS = K-SADS Mania Rating Scale  ^g^KDRS = K-SADS Depression Rating Scale | | | | | |

***Table S2. Between-group differences of RD in the 4 main groups (BD, OBP, OCP, and OHP).***

| **Tract** | **Nodes^a^**  **in cluster** | **Comparisons** | **Mean Diff.^b^** | **SE^c^** | **p-value** | **FDR^d^**  **Corrected**  **q-value^b^** |
| --- | --- | --- | --- | --- | --- | --- |
| Left ATR^e^ | **75-98** | **BD > OBP** | 6.371E-5 | 0.000 | <0.001 | **<0.001** |
|  |  | **BD > OCP** | 4.058E-5 | 0.000 | 0.011 | **0.044** |
|  |  | BD > OHP | 2.310E-05 | 0.000 | 0.132 | 0.211 |
|  |  | OBP < OCP | 2.313E-5 | 0.000 | 0.119 | 0.204 |
|  |  | **OBP < OHP** | 4.061E-5 | 0.000 | 0.005 | **0.024** |
|  |  | OCP < OHP | 1.748E-05 | 0.000 | 0.210 | 0.296 |
| Right ATR | **83-98** | BD > OBP | 3.097E-05 | 0.000 | 0.054 | 0.129 |
|  |  | BD > OCP | 1.668E-05 | 0.000 | 0.291 | 0.388 |
|  |  | BD > OHP | 7.786E-06 | 0.000 | 0.610 | 0.637 |
|  |  | OBP < OCP | 1.429E-05 | 0.000 | 0.333 | 0.421 |
|  |  | OBP < OHP | 2.318E-05 | 0.000 | 0.106 | 0.196 |
|  |  | OCP < OHP | 8.897E-06 | 0.000 | 0.522 | 0.569 |
| Right CB^f^ | **15-26** | **BD > OBP** | 3.997E-5 | 0.000 | 0.001 | **0.006** |
|  |  | **BD > OCP** | 2.963E-5 | 0.000 | 0.016 | **0.048** |
|  |  | BD > OHP | 2.601E-5 | 0.000 | *0.029* | 0.077 |
|  |  | OBP < OCP | 1.033E-05 | 0.000 | 0.363 | 0.436 |
|  |  | OBP < OHP | 1.396E-05 | 0.000 | 0.205 | 0.296 |
|  |  | OCP < OHP | 3.628E-06 | 0.000 | 0.735 | 0.735 |
| Right CB | **50-61** | **BD > OBP** | 5.155E-5 | 0.000 | <0.001 | **<0.001** |
|  |  | **BD > OCP** | 3.184E-5 | 0.000 | 0.014 | **0.048** |
|  |  | BD > OHP | 1.049E-05 | 0.000 | 0.395 | 0.451 |
|  |  | OBP < OCP | 1.971E-05 | 0.000 | 0.100 | 0.196 |
|  |  | **OBP < OHP** | 4.106E-5 | 0.000 | 0.001 | **0.006** |
|  |  | OCP < OHP | 2.135E-05 | 0.000 | 0.059 | 0.129 |
| ^a^ Each tract was divided into 98 nodes  ^b^ Mean Diff = Mean Difference  ^c^ SE = Standard Error  ^d^ q-values ≤ 0.05 are reported in bold characters  ^e^ ATR = anterior thalamic radiation  ^f^ CB = cingulum bundle | | | | | | |

***Table S3. Between-group differences in FA in BD,*** *healthy* ***OBP*** *and* ***OCP,*** *non-BD OBP and OCP****, and OHP.***

| **Tract** | **Nodes^a^**  **in cluster** | **Comparisons** | **Mean Diff.^b^** | **SE^c^** | **p-value** | **FDR^d^**  **corrected** |
| --- | --- | --- | --- | --- | --- | --- |
| Left ATR^e^ | **75-98** | **healthy OBP and OCP > BD** | 0.041 | 0.008 | <0.001 | **<0.001** |
|  |  | healthy OBP and OCP > non-BD OBP and OCP | 0.013 | 0.007 | 0.078 | 0.094 |
|  |  | **healthy OBP and OCP > OHP** | 0.027 | 0.007 | <0.001 | **0.001** |
|  |  | **non-BD OBP and OCP** **> BD** | 0.028 | 0.008 | 0.001 | **0.003** |
|  |  | non-BD OBP and OCP > OHP | 0.013 | 0.007 | 0.067 | 0.089 |
| Right ATR | **83-98** | **healthy OBP and OCP > BD** | 0.038 | 0.006 | <0.001 | **<0.001** |
|  |  | **healthy OBP and OCP > non-BD OBP and OCP** | 0.016 | 0.006 | 0.011 | **0.024** |
|  |  | **healthy OBP and OCP > OHP** | 0.028 | 0.005 | <0.001 | **<0.001** |
|  |  | **non-BD OBP and OCP > BD** | 0.022 | 0.007 | 0.001 | **0.003** |
|  |  | non-BD OBP and OCP > OHP | 0.012 | 0.006 | 0.035 | 0.060 |
| Right CB^f^ | **15-26** | **healthy OBP and OCP > BD** | 0.025 | 0.011 | 0.024 | **0.048** |
|  |  | non-BD OBP and OCP > healthy OBP and OCP | 0.021 | 0.010 | 0.044 | 0.066 |
|  |  | OHP > healthy OBP and OCP | 0.003 | 0.009 | 0.757 | 0.790 |
|  |  | **non-BD OBP and OCP > BD** | 0.046 | 0.011 | <0.001 | **0.001** |
|  |  | non-BD OBP and OCP > OHP | 0.018 | 0.010 | 0.072 | 0.091 |
| Right CB | **50-61** | **healthy OBP and OCP > BD** | 0.034 | 0.013 | 0.010 | **0.024** |
|  |  | non-BD OBP and OCP > healthy OBP and OCP | 0.005 | 0.013 | 0.693 | 0.756 |
|  |  | **healthy OBP and OCP > OHP** | 0.031 | 0.011 | 0.007 | **0.021** |
|  |  | non-BD OBP and OCP > BD | 0.029 | 0.014 | 0.039 | 0.062 |
|  |  | non-BD OBP and OCP > OHP | 0.026 | 0.012 | 0.033 | 0.060 |
| ^a^ Each tract was divided into 98 nodes  ^b^ Mean Diff = Mean Difference  ^c^ SE = Standard Error  ^d^ q-values ≤ 0.05 are reported in bold characters  ^e^ ATR = anterior thalamic radiation  ^f^ CB = cingulum bundle | | | | | | |

***Table S4. Differences of FA between Converters and the 4 main groups (BD, OBP, OCP, OHP).***

| **Tract** | **Nodes^a^**  **in cluster** | **Comparisons** | **Mean Diff.^b^** | **SE^c^** | **p-value** | **FDR^d^**  **corrected**  **q-value^b^** |
| --- | --- | --- | --- | --- | --- | --- |
| Left ATR^e^ | **75-98** | Converters > BD | 0.018 | 0.014 | 0.207 | 0.345 |
|  |  | Converters < OBP | 0.020 | 0.014 | 0.139 | 0.277 |
|  |  | Converters < OCP | 0.016 | 0.013 | 0.243 | 0.374 |
|  |  | Converters > OHP | 0.003 | 0.013 | 0.826 | 0.870 |
| Right ATR | **83-98** | Converters > BD | 0.007 | 0.011 | 0.572 | 0.689 |
|  |  | **Converters < OBP** | **0.027** | **0.011** | **0.016** | **0.045** |
|  |  | Converters < OCP | *0.021* | *0.011* | *0.066* | *0.165* |
|  |  | Converters < OHP | 0.003 | 0.011 | 0.805 | 0.870 |
| Right CB^f^ | **15-26** | Converters > BD | 0.011 | 0.020 | 0.586 | 0.689 |
|  |  | Converters < OBP | 0.029 | 0.019 | 0.130 | 0.267 |
|  |  | Converters < OCP | 0.018 | 0.019 | 0.365 | 0.493 |
|  |  | Converters < OHP | 0.017 | 0.019 | 0.370 | 0.493 |
| Right CB | **50-61** | Converters > BD | 0.009 | 0.023 | 0.692 | 0.787 |
|  |  | Converters < OBP | 0.030 | 0.023 | 0.182 | 0.317 |
|  |  | Converters < OCP | 0.013 | 0.023 | 0.576 | 0.699 |
|  |  | Converters > OHP | 0.008 | 0.022 | 0.708 | 0.787 |
| ^a^ Each tract was divided into 98 nodes  ^b^ Mean Diff = Mean Difference  ^c^ SE = Standard Error  ^d^ q-values ≤ 0.05 are reported in bold characters  ^e^ ATR = anterior thalamic radiation  ^f^ CB = cingulum bundle | | | | | | |

***Table S5. Relationships between mean FA and symptom severity across BD, OBP, and OCP^a^***

| **Left ATR^b^ (nodes 75-98)** | | | |
| --- | --- | --- | --- |
|  | ***rho*** | ***p-value*** | ***FDR corrected^c^*** |
| SCARED^d^ – parent version | -0.357 | 0.002 | **0.028** |
| SCARED – child version | -0.445 | <0.001 | **0.003** |
| CALS^e^ – parent version | -0.347 | 0.003 | **0.029** |
| CALS – child version | -0.550 | <0.001 | **<0.001** |
| MFQ^f^ – parent version | -0.302 | 0.010 | 0.063 |
| MFQ – child version | -0.385 | 0.001 | **0.018** |
| KMRS^g^ | -0.409 | <0.001 | **<0.001** |
| KDRS^h^ | -0.374 | 0.001 | **0.018** |
| **Right ATR (nodes 83-98)** | | | |
|  | ***rho*** | ***p-value*** | ***FDR corrected*** |
| SCARED – parent version | -0.350 | 0.003 | **0.029** |
| SCARED – child version | -0.367 | 0.001 | **0.022** |
| CALS – parent version | -0.235 | 0.047 | 0.179 |
| CALS – child version | -0.446 | <0.001 | **0.003** |
| MFQ – parent version | -0.173 | 0.147 | 0.321 |
| MFQ – child version | -0.233 | 0.047 | 0.179 |
| KMRS | -0.387 | 0.001 | **0.018** |
| KDRS | -0.232 | 0.049 | 0.181 |
| **Anterior cluster of right CB^i^ (nodes 15-26)** | | | |
|  | ***rho*** | ***p-value*** | ***FDR corrected*** |
| SCARED – parent version | -0.267 | 0.023 | 0.119 |
| SCARED – child version | -0.193 | 0.103 | 0.269 |
| CALS – parent version | -0.346 | 0.003 | **0.029** |
| CALS – child version | -0.236 | 0.044 | 0.179 |
| MFQ – parent version | -0.339 | 0.004 | **0.032** |
| MFQ – child version | -0.087 | 0.467 | 0.640 |
| KMRS | -0.078 | 0.514 | 0.672 |
| KDRS | -0.064 | 0.591 | 0.740 |
| **Middle cluster of right CB (nodes 50-61)** | | | |
|  | ***rho*** | ***p-value*** | ***FDR corrected*** |
| SCARED – parent version | -0.210 | 0.077 | 0.246 |
| SCARED – child version | -0.321 | 0.006 | **0.048** |
| CALS – parent version | -0.191 | 0.108 | 0.269 |
| CALS – child version | -0.196 | 0.097 | 0.269 |
| MFQ – parent version | -0.043 | 0.720 | 0.798 |
| MFQ – child version | -0.085 | 0.475 | 0.645 |
| KMRS | -0.140 | 0.238 | 0.427 |
| KDRS | -0.198 | 0.093 | 0.269 |
| ^a^ Children of bipolar parents with bipolar disorder (BD); offspring of bipolar parents (OBP); offspring of comparison parents (OCP)  ^b^ ATR = anterior thalamic radiation  ^c^ q-values < 0.05 are reported in bold characters, p-values uncorrected < 0.05 are reported in italic characters  ^d^ SCARED = Screen for Childhood Anxiety and Related Disorders  ^e^ CALS = Child Affect Lability Scale  ^f^ MFQ = Mood and Feelings Questionnaire  ^g^ KMRS = K-SADS Mania Rating Scale  ^h^ KDRS = K-SADS Depression Rating Scale  ^i^ CB = cingulum bundle | | | |

***Table S6. Relationships between mean FA and the age of BD onset, and illness duration in BD***

|  | **Left ATR^a^**  (nodes 75-98) | | | **Right ATR**  (nodes 83-98) | | | **Anterior Right CB^b^**  (nodes 15-26) | | | **Middle Right CB**  (nodes 50-61)  **50-61** | | |
| --- | --- | --- | --- | --- | --- | --- | --- | --- | --- | --- | --- | --- |
|  | **rho** | p | **FDR corr.^c^** | **rho** | p | **FDR corr.** | **rho** | p | **FDR corr.** | **rho** | p | **FDR corr.** |
| Age of onset | -0.355 | 0.114 | 0.269 | -0.196 | 0.393 | 0.577 | -0.344 | 0.127 | 0.282 | -0.572 | 0.007 | **0.049** |
| Illness duration | 0.438 | *0.047* | 0.179 | 0.278 | 0.223 | 0.411 | 0.382 | 0.088 | 0.269 | 0.151 | 0.515 | 0.672 |
| ^a^ ATR = anterior thalamic radiation  ^b^  CB = cingulum bundle  ^c^ FDR corrected, q-values < 0.05 are reported in bold characters | | | | | | | | | | | | |

| ***Table S7. Effects of group on mean FA in each tract of interest using SES as a covariate instead of IQ.*** | | | |
| --- | --- | --- | --- |
| **Tract** | **F** | **p-value** | **FDR corrected q-value** |
| Forceps minor | 0.242 | 0.867 | 0.867 |
| Left ATR | **5.453** | **0.002** | **0.007** |
| Right ATR | **6.739** | **< 0.001** | **< 0.001** |
| Left CB | 2.793 | 0.044 | 0.062 |
| Right CB | **4.878** | **0.003** | **0.007** |
| Left UF | 2.863 | 0.041 | 0.062 |
| Right UF | 2.288 | 0.083 | 0.097 |
| q-values ≤ 0.05 are reported in bold characters  ATR = anterior thalamic radiation  CB = cingulum bundle  UF = uncinate fasciculus | | | |

Fig. S1. 3D visualization of the white matter tracts showing group differences across 5 groups (BD, OBP, OCP, OHP, and Converters). Bar plots represent the mean FA with a main effect of group (FDR-corrected) in 4 clusters. Brackets show the significant difference between groups (q < 0.05). Error bars represent the standard deviations. BD - offspring with BD; OBP - offspring of bipolar parents; OCP - offspring of comparison parents; OHP - healthy offspring of healthy parents. A. Left Anterior Thalamic Radiation. B. Right Anterior Thalamic Radiation. C. Anterior cluster of the Right Cingulum Bundle. D. Middle cluster of the Right Cingulum Bundle.


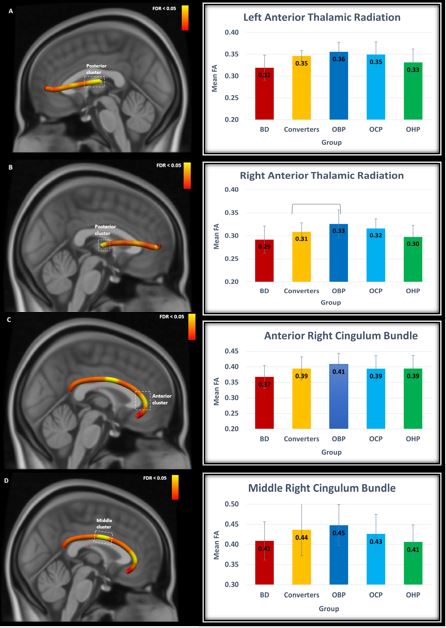

Supplement: Supplementary file 1 — Differentiating white matter measures that protect against vs. predispose to Bipolar Disorder and other psychopathology in at-risk youth [file 41386_2021_1088_MOESM1_ESM.docx]
